# SNPs in the TGF-β Signaling Pathway Are Associated with Increased Risk of Brain Metastasis in Patients with Non–Small-Cell Lung Cancer

**DOI:** 10.1371/journal.pone.0051713

**Published:** 2012-12-17

**Authors:** Qianxia Li, Huanlei Wu, Bei Chen, Guangyuan Hu, Liu Huang, Kai Qin, Yu Chen, Xianglin Yuan, Zhongxing Liao

**Affiliations:** 1 Department of Oncology, Tongji Hospital, Huazhong University of Science and Technology, Wuhan, Hubei Province, China; 2 Department of Radiation Oncology, The University of Texas MD Anderson Cancer Center, Houston, Texas, United States of America; 3 Department of Electrocardiographic Room, Hubei Provincial Tumor Hospital, Wuhan, Hubei Province, China; Dartmouth, United States of America

## Abstract

**Purpose:**

Brain metastasis (BM) from non-small cell lung cancer (NSCLC) is relatively common, but identifying which patients will develop brain metastasis has been problematic. We hypothesized that genotype variants in the TGF-β signaling pathway could be a predictive biomarker of brain metastasis.

**Patients and Methods:**

We genotyped 33 SNPs from 13 genes in the TGF-β signaling pathway and evaluated their associations with brain metastasis risk by using DNA from blood samples from 161 patients with NSCLC. Kaplan-Meier analysis was used to assess brain metastasis risk; Cox hazard analyses were used to evaluate the effects of various patient and disease characteristics on the risk of brain metastasis.

**Results:**

The median age of the 116 men and 45 women in the study was 58 years; 62 (39%) had stage IIIB or IV disease. Within 24 months after initial diagnosis of lung cancer, brain metastasis was found in 60 patients (37%). Of these 60 patients, 16 had presented with BM at diagnosis. Multivariate analysis showed the GG genotype of *SMAD6*: rs12913975 and TT genotype of *INHBC*: rs4760259 to be associated with a significantly higher risk of brain metastasis at 24 months follow-up (hazard ratio [HR] 2.540, 95% confidence interval [CI] 1.204–5.359, *P* = 0.014; and HR 1.885, 95% CI 1.086–3.273, *P* = 0.024), compared with the GA or CT/CC genotypes, respectively. When we analyzed combined subgroups, these rates showed higher for those having both the GG genotype of *SMAD6*: rs12913975 and the TT genotype of *INHBC*: rs4760259 (HR 2.353, 95% CI 1.390–3.985, *P* = 0.001).

**Conclusions:**

We found the GG genotype of *SMAD6*: rs12913975 and TT genotype of *INHBC*: rs4760259 to be associated with risk of brain metastasis in patients with NSCLC. This finding, if confirmed, can help to identify patients at high risk of brain metastasis.

## Introduction

More 150,000 patients with cancer are diagnosed with brain metastasis each year [1], with the lung being the most common primary site for secondary BM [2], [3]. Improved RT techniques and the increased use of combined-modality therapy have reduced distant metastases and significantly improved survival. However, it has shown to be associated with increased rates of overall brain failure [4]. The outcome of the diagnosis of brain metastases is dismal. Even for young patients with good performance status and controlled extra-cranial disease, the median survival time for patients after the development of BM is only about 7 months [5]. Means of preventing the development of BM are therefore urgently sought. For example, prophylactic cranial irradiation (PCI) has a clearly defined role in the treatment of high-risk patients with acute lymphocytic leukemia and patients with small-cell lung cancer (SCLC) [6]. These are radiosensitive tumors where moderate doses of radiation can be employed and result in significant improvements in intracranial control in addition to overall survival, and therefore PCI is considered standard of care. Perhaps patients with non-small cell lung cancer (NSCLC) as well. Prior randomized, controlled trials and several prospective studies without brain primary end points and retrospective studies evaluating PCI for NSCLC have consistently shown a decrease and/or delay in BM with PCI [4], [7], [8], [9]. But PCI has not become part of standard management for LA-NSCLC because of concern for long-term toxicity and lack of a proven survival benefit. It is unclear as to whether this is secondary to failure of identifying the cohort best suited for prevention. The authors of this paper hypothesized that among NSCLC patients of stage I-IV may exist a group of patients at high risk of presenting BM. This group should be identified in order to serve as target for future studies of PCI application in NSCLC and avoid side effects for those who at low risk of presenting BM.

Defining the cohort of high-risk patients is difficult, because it is dependent on reports that often have conflicting results. Pretreatment factors that predict for high rates of BM include histology, extent of disease, and young age. However, not all studies have shown a significant correlation [10], [11], [12]. The expression levels of three genes, *CDH2* (N-cadherin), *KIFC1*, and *FALZ*, was found in one study to be highly predictive of BM in early and advanced lung cancer [13]. The expression levels of genes can be effect by other factors and not so precise which seriously limits this approach for risk prediction. Rarely study addressing the question about the association between polymorphisms and brain failure. Moreover, the heterogeneity and genetic complexity of NSCLC make it unlikely that any single SNP would be sufficient to confer the risk of BM. Rather, studying multiple SNPs in signaling pathways that regulate cell proliferation and migration may be a more powerful way of pinpointing the genes and polymorphisms involved in conferring risk of BM. One such pathway is that of transforming growth factor-β (TGF-β).

The TGF-β superfamily comprises TGF-βs, bone morphogenetic proteins, activins, and related proteins. TGF-β signaling pathways have diverse effects on cell proliferation, morphogenesis, migration, extracellular matrix production, and apoptosis. In particular, TGF-β suppresses early-stage tumor development by virtue of its potent growth inhibitory effect, but becomes a pro-oncogenic factor that stimulates tumor cell growth and invasiveness at later stages of tumorigenesis [14], [15]. Tumor cells can escape the antiproliferative effects of TGF-β by acquiring mutations in components of signaling pathways or by selectively disrupting TGF-β signaling. The epithelial-to-mesenchymal transition (EMT) is associated with cellular acquisition of motility and invasive properties that promote the formation of distant metastasis [16]. A variety of other mechanisms, including changes in expression of cell-cell adhesion molecules and secretion of metalloproteinases, also contribute to the metastatic process [17].

Given the prominent role of the TGF-β pathway in maintaining cellular function and the effect of its disruption on distant metastasis, common genetic variations in this pathway may emerge as potential predictors of BM risk. In this study, we tested the hypothesis that common genetic variants in the TGF-β pathway are associated with BM risk, and we attempted to identify subgroups of patients with NSCLC who are at particularly high risk of developing BM.

## Patients and Methods

### Patient Population

Subjects in this study were selected from a total of 201 patients with lung cancer who had been treated at either the Tongji Hospital Cancer Center or the Hubei Provincial Tumor Hospital in 2008–2009 who also had blood samples available for analysis. All study participants provided written informed consent before blood samples were collected. The study was approved by the Ethics Committee of Tongji Medical College. Of these 201 patients, 190 had documentation having undergone complete disease staging, and 161 had pathologically confirmed NSCLC. These 161 patients were the basis of this analysis. Clinical data were obtained from patients’ files. Disease had been staged in terms of the tumor/node/metastasis system in the sixth (2002) edition of the American Joint Committee on Cancer staging manual. The diagnosis of BM was based on computed tomography or magnetic resonance imaging records. The smoking status includes current, former, or never smoker. Former smokers were defined as individuals who had successfully ceased smoking for at least 1 year at the time of study registration. Never smokers were defined as individuals who had <20 total cigarettes during their lifetime [18]. The time to BM was the interval from the date of NSCLC diagnosis to the date of BM diagnosis. The follow-up time was the interval from NSCLC diagnosis to death or to the last hospital visit.

### Genotyping Methods

The procedures used to select SNPs in the TGF-β pathway have been described previously [19]. Briefly, we used databases at Gene Oncology (http://www.geneontology.org) and the National Center for Biotechnology Information (NCBI)’s Gene database (http://www.ncbi.nlm.nih.gov/gene) and related literature to identify all functional single nucleotide polymorphisms (SNPs) of the genes in TGF-β signaling pathways with a minor allele frequency of more than 0.05 in a Chinese population. We selected 33 SNPs in 13 genes related to TGF-β pathways that were either located in the promoter untranslated region or coding region of the gene or had been previously reported as being associated with survival, lung cancer, or general metastasis (**Table 1**). Genomic DNA was isolated from peripheral blood lymphocytes by using the QuickGene DNA whole blood kit S (Fuji Film) and stored at –80°C until use. Thirty-two of the SNPs were genotyped by using MALDI-TOF mass spectrophotometry to detect allele-specific primer extension products with the MassARRAY platform (Sequenom, Inc.). Assay data were analyzed using Sequenom TYPER software (version 4.0). The individual call rate threshold was at least 95%. The 33^rd^ SNP (*TGFB1:* rs1800470) was genotyped by using the TaqMan assay [20].

**Table 1:** Genes and single-nucleotide polymorphisms (SNPs) selected for analysis.

Gene(number of SNPs)	SNP	Allelic change
*TGFB1* (3)	rs 4803455	A>C
	rs 1800469	C>T
	rs 1800470	C>T
*BMP1* (2)	rs 3857979	C>T
	rs 7838961	A>G
*BMP2* (1)	rs 235756	C>T
*BMP4* (2)	rs 17563	C>T
	rs 8014071	G>T
*INHBC* (1)	rs 4760259	C>T
*TGFBR1* (3)	rs 10819638	C>T
	rs 6478974	A>T
	rs 10733710	A>G
*ACVR2A* (1)	rs 1424954	A>G
*SMAD1* (1)	rs 11939979	A>C
*SMAD3* (7)	rs 4776342	A>G
	rs 12102171	A>C
	rs 6494633	C>T
	rs 11632964	C>T
	rs 750766	A>G
	rs 4776343	A>G
	rs 11071938	C>T
*SMAD4* (6)	rs 948588	A>G
	rs 12456284	A>G
	rs 7244227	A>G
	rs 12455792	C>T
	rs 12958604	A>G
	rs 10502913	A>G
*SMAD6* (3)	rs 12913975	A>G
	rs 12906898	A>G
	rs 4776318	A>C
*SMAD7* (1)	rs 7227023	A>G
*SMAD8* (2)	rs 7333607	A>G
	rs 511674	A>G

NOTE. A total of 33 SNPs from 13 TGF-β pathway-related genes were genotyped.

### Statistical Analysis

This analysis was undertaken after all patients had been potentially observed for a minimum of 24 months. Patients were grouped according to genotype. Statistical analysis was performed using SPSS (version 16.0) software. Cox proportional hazards model was used to calculate hazard ratio (HR) and 95% confidence intervals (CIs) for multivariate survival analyses, while adjusting for sex, age, disease stage, tumor histology, Karnofsky performance status (KPS), and smoking status. Kaplan-Meier plots and log rank tests were used to estimate the effect of genotype on BM risk. Likelihood ratio tests were used for each multivariate Cox regression to assess goodness-of-fit. A *P* value of ≤0.05 was considered to indicate statistical significance in two-sided *t* tests.

## Results

### Patient Characteristics

Characteristics of the 161 patients (116 men and 45 women) are shown in **Table 2**. The median age was 58 years (range, 32 to 80 years); 61% had stage ≤IIIA disease; 60% had adenocarcinoma, and 54% had smoked tobacco (71.6% in male and 8.9% in female).

**Table 2:** Patient and disease characteristics and their association with brain metastasis.

Characteristic	No. ofPatients (%)	HR	Univariate Analysis(95% CI)	*P* Value	HR	Multivariate Analysis (95% CI)	*P* Value
Sex							
Female	45 (28)	1.000			1.000		
Male	116 (72)	0.827	0.480–1.425	0.493	0.890	0.457–1.734	0.732
Age, years							
≥60 years		1.000			1.000		
<60 years		1.185	0.709–1.980	0.517	0.990	0.585–1.674	0.970
Median (range)	58 (32–80)						
Disease stage at diagnosis							
I, II, IIIA	99(61)	1.000			1.000		
IIIB, IV	62(39)	3.796	2.247–6.412	0.000	3.786	2.228–6.431	0.000
Tumor histology							
Squamous cell	51(32)	1.000			1.000		
Adenocarcinoma	97(60)	1.968	1.060–3.656	0.032	1.610	0.828–3.129	0.161
NSCLC, NOS	13 (8)	0.895	0.255–3.140	0.862	0.945	0.261–3.423	0.931
KPS							
>80	22 (14)	1.000			1.000		
80	87 (54)	1.560	0.655–3.717	0.315	1.046	0.425–2.577	0.921
<80	52 (32)	1.538	0.617–3.830	0.355	1.277	0.502–3.245	0.608
Smoking status/Tobacco use							
current	62 (38)	1.000			1.000		
former	25 (16)	1.965	0.970–3.982	0.061	1.677	0.798–3.523	0.172
never	74 (46)	1.261	0.704–2.258	0.436	0.959	0.479–1.921	0.906

NOTE. Multivariate analyses were adjusted for all factors listed in Table.

Abbreviations: HR, hazard ratio; CI, confidence interval; NSCLC NOS, non-small cell lung cancer, not otherwise specified; KPS, Karnofsky performance status.

### Brain Metastasis and Genotypes

The median time from NSCLC diagnosis to detection of BM was 7.5 months (range, 0 to 23 months). The median time was 10 months when patients who presented with BM were excluded. Associations between patient- and tumor-related characteristics and BM by univariate and multivariate analyses are shown in **Table 2**. As expected, disease stage was associated with BM, with patients having stage IIIB or stage IV disease at higher risk of BM (*P*<0.010). And patients with adenocarcinoma were associated with higher risk of BM by Cox hazard analyses (*P* = 0.032). However, the smoking status has no association with BM risk in this population.

**Figure 1:**
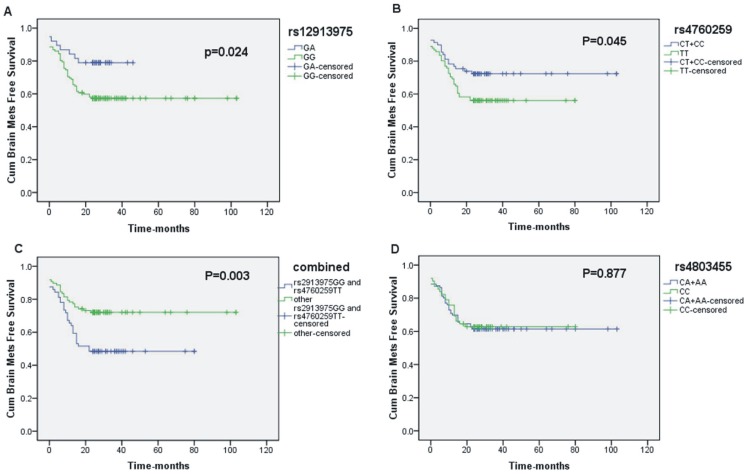
Kaplan-Meier curves showing brain metastasis-free survival among patients with non-small cell lung cancer. Patients were stratified by genotypes. (A) *SMAD6*: rs12913975; (B) *INHBC*: rs4760259; (C) combined; (D) *TGFB1*: rs4803455. The GG genotype for rs12913975 and the TT genotype for rs4760259 were associated with significantly lower cumulative brain metastasis-free survival compared with the other genotypes.

**Figure 1** illustrate cumulative BM-free survival rates for all patients according to genotype. These rates remained lower for those with either the GG genotype of *SMAD6*: rs12913975 (*P* = 0.024, **Figure 1A**) or the TT genotype of *INHBC*: rs4760259 (*P* = 0.045, **Figure 1B**). When we analyze combined subgroups, these rates showed lower for those having both the GG genotype of *SMAD6*: rs12913975 and the TT genotype of *INHBC*: rs4760259 (*P* = 0.003, **Figure 1C**). Other 31 SNPs in the TGF-β pathway in **Table 1** were also analyzed, but no significant correlation was found (*P* = 0.877, **Figure 1D** for *TGFB1*: rs4803455; date of other 30 selected SNPs not shown).

**Table 3** and **4** lists the findings of Kaplan-Meier analyses of BM incidence according to genotype at 24 months from diagnosis. In general, BM developed more often in patients with the GG genotype of *SMAD6*: rs12913975 (43%) or the TT genotype of *INHBC*: rs4760259 (44%) compared with the GA (21%) or CT/CC genotypes (27%). These associations between genotype and BM were statistically significant for both subgroups. When we analyze combined subgroups, we can see having both GG (rs12913975) and TT (rs4760259) show a higher probability of BM (52% vs 28%, *P* = 0.003, **Table 4**). Multivariate Cox proportional hazard analyses showed the GG genotype of *SMAD6*: rs12913975 and TT genotype of *INHBC*: rs4760259 to be associated with a significantly higher risk of brain metastasis (HR 2.540, 95% CI 1.204–5.359, *P* = 0.014; and HR 1.885, 95% CI 1.086–3.273, *P* = 0.024, respectively), after adjustment for stage, histology, age, and smoking status. When analyze combined subgroups, these rates showed higher for those having both the GG genotype of *SMAD6*: rs12913975 and the TT genotype of *INHBC*: rs4760259 (HR 2.353, 95% CI 1.390–3.985, *P* = 0.001). Moreover, when we repeated the analysis excluding the patients who had presented with BM at the diagnosis of NSCLC, the association between BM and both genotypes remained significant (**Table 3** and **4**). Similar analyses of the other 31 selected SNPs showed no associations between any other genotype and the incidence of BM (**Table 5** and **6**).

**Table 3:** Associations between genotypes and brain metastases.

Polymorphisms andGenotypes	No. ofPatients (All)	No. ofEvents (%)	HR	95% CI	*P* Value	No. of Patients withoutBM at Diagnosis	No. of Events(%)	HR	95% CI	*P* Value
*SMAD6*: rs12913975										
GA	38	8 (21)	1.000			36	6 (17)	1.000		
GG	122	52 (43)	2.540	1.204–5.359	0.014	108	38 (35)	2.577	1.086–6.117	0.032
*INHBC*: rs4760259										
CT or CC	69	19 (27)	1.000			64	14 (22)	1.000		
TT	91	40 (44)	1.885	1.086–3.273	0.024	81	30 (37)	1.961	1.032–3.727	0.040

NOTE. Multivariate analyses in this table were adjusted for Stage, Histology, Age, and Smoking status. Similar results were obtained when multivariate analyses were adjusted for all the factors listed in Table1 (data not shown).

Abbreviations: HR, hazard ratio; CI, confidence interval; BM, brain metastases.

**Table 4:** Associations between genotypes and brain metastases.

Polymorphisms and Genotypes	No. of Patients(All)	No. of Events(%)	HR	95% CI	*P* Value	No. of Patients WithoutBM at Diagnosis	No. of Events(%)	HR	95% CI	*P* Value
Other	97	27 (28)	1.000			89	19 (21)	1.000		
GG (rs12913975) And TT (rs4760259)	64	33 (52)	2.353	1.390–3.985	0.001	56	25 (45)	2.648	1.424–4.924	0.002

NOTE. Multivariate analyses in this table were adjusted for Stage, Histology, Age, and Smoking status. Similar results were obtained when multivariate analyses were adjusted for all the factors listed in Table1 (data not shown).

Abbreviations: HR, hazard ratio; CI, confidence interval; BM, brain metastas.

**Table 5:** Associations between genotypes and brain metastases (the other 31 selected SNPs).

Polymorphisms and Genotypes		No. of Patients (All)	No. of Events (%)		HR		95% CI		*P* value	
*TGFB1*: rs4803455										
CA or AA		96	37(39)		1.000					
CC		62	23(37)		1.137		0.670–1.929		0.634	
*TGFB1*: rs1800469										
CT or CC		118	45(38)		1.000					
TT		41	15(37)		1.222		0.674–2.216		0.510	
*TGFB1*: rs1800470										
CT or TT		112	42(38)		1.000					
CC		45	17(38)		1.180		0.662–2.103		0.575	
*BMP1*: rs3857979										
CT or TT		72	23(32)		1.000					
CC		89	37(42)		1.298		0.768–2.196		0.330	
*BMP1*: rs7838961										
GA or GG		75	26(35)		1.000					
AA		86	34(40)		1.165		0.693–1.959		0.564	
*BMP2*: rs235756										
TC or CC		48	19(40)		1.000					
TT		113	41(37)		0.932		0.538–1.613		0.800	
*BMP4*: rs17563										
TC or CC		88	31(35)		1.000					
TT		73	29(40)		0.920		0.541–1.565		0.759	
*BMP4*: rs8014071										
AG or GG		93	38(41)		1.000					
AA		65	22(34)		0.890		0.524–1.513		0.667	
*TGFBR1*: rs10819638										
CT or TT		102	37(36)		1.000					
CC		59	23(39)		1.394		0.812–2.393		0.229	
*TGFBR1*: rs6478974										
TA or AA		86	29(34)		1.000					
TT		75	31(41)		1.006		0.602–1.682		0.981	
*TGFBR1*: rs10733710										
GA or AA		69	30(44)		1.000					
GG		91	29(32)		0.690		0.407–1.171		0.169	
*ACVR2A*: rs1424954										
GA or GG		111	42(38)		1.000					
AA		49	18(37)		1.004		0.572–1.762		0.988	
*SMAD1*: rs11939979										
CA or CC		63	24(38)		1.000					
AA		95	35(37)		0.947		0.552–1.624		0.843	
*SMAD3*:rs4776342										
AG or AA		112	42(38)		1.000					
GG		49	18(37)		1.060		0.607–1.852		0.836	
*SMAD3*:rs12102171										
CT or TT		90	34(38)		1.000					
CC		71	26(37)		1.156		0.685–1.952		0.587	
*SMAD3*:rs6494633										
CT		33	11(33)		1.000					
CC		128	49(38)		0.801		0.404–1.588		0.526	
*SMAD3*:rs11632964										
TC or TT		94	37(39)		1.000					
CC		67	23(34)		0.755		0.445–1.282		0.298	
*SMAD3*:rs750766										
AG or AA		98	35(36)		1.000					
GG		62	25(40)		1.215		0.721–2.048		0.465	
*SMAD3*:rs4776343										
AG		14	6(43)		1.000					
GG		147	54(37)		0.931		0.395–2.192		0.869	
*SMAD3*:rs11071938										
TC or TT		72	27(38)		1.000					
CC		89	33(37)		1.077		0.646–1.795		0.776	
*SMAD4*:rs948588										
GA or AA		17	9(53)		1.000					
GG		144	51(35)		0.740		0.359–1.523		0.413	
*SMAD4*:rs12456284										
AG or GG		93	34(37)		1.000					
AA		65	24(37)		0.845		0.494–1.444		0.537	
*SMAD4*:rs7244227										
AG or GG		109	41(38)		1.000					
AA		50	19(38)		0.905		0.521–1.571		0.722	
*SMAD4*:rs12455792										
CT or TT		110	45(41)		1.000					
CC		49	15(31)		0.612		0.338–1.107		0.104	
*SMAD4*:rs12958604										
AG or GG		125	47(38)		1.000					
AA		36	13(36)		0.828		0.446–1.540		0.552	
*SMAD4*:rs10502913										
AG or AA		87	32(37)		1.000					
GG		74	28(38)		0.920		0.549–1.541		0.920	
*SMAD6*: rs12906898										
AG or AA	37		13(35)	1.000						
GG	122		47(39)	1.297		0.688–2.444		0.421		
*SMAD6*: rs4776318										
CA or AA	57		20(35)	1.000						
CC	103		40(39)	1.161		0.662–2.037		0.603		
*SMAD7*: rs7227023										
GA	8		4(50)	1.000						
GG	153		56(37)	0.808		0.284–2.301		0.690		
*SMAD8*: rs7333607										
AG	29		12(41)	1.000						
AA	128		48(38)	0.942		0.497–1.786		0.855		
*SMAD8*: rs511674										
GA	30		10(33)	1.000						
AA	131		50(38)	1.341		0.678–2.653		0.399		

NOTE. Multivariate analyses in this table were adjusted for Stage, Histology, Age, and Smoking status. Similar results were obtained when multivariate analyses were adjusted for all the factors listed in Table 1 (data not shown).

Abbreviations: HR, hazard ratio; CI, confidence interval.

**Table 6:** Associations between genotypes and brain metastases, excluding those with brain metastases at initial diagnosis of non-small cell lung cancer (the other 31 selected SNPs).

Polymorphisms and Genotypes	No. of Patients (All)	No. of Events (%)	HR	95% CI	*P* value		
*TGFB1*: rs4803455							
CA or AA	85	26(31)	1.000				
CC	57	18(32)	1.257	0.680–2.326	0.466		
*TGFB1*: rs1800469							
CT or CC	105	32(31)	1.000				
TT	38	12(32)	1.335	0.679–2.628	0.402		
*TGFB1*: rs1800470							
CT or TT	100	30(30)	1.000				
CC	41	13(32)	1.187	0.608–2.320	0.615		
*BMP1*: rs3857979							
CT or TT	67	18(27)	1.000				
CC	78	26(33)	1.249	0.682–2.289	0.472		
*BMP1*: rs7838961							
GA or GG	69	20(29)	1.000				
AA	76	24(32)	1.138	0.622–2.083	0.676		
*BMP2*: rs235756							
TC or CC	41	12(29)	1.000				
TT	104	32(31)	1.108	0.569–2.158	0.762		
*BMP4*: rs17563							
TC or CC	77	20(26)	1.000				
TT	68	24(35)	1.312	0.707–2.435	0.389		
*BMP4*: rs8014071							
AG or GG	81	26(32)	1.000				
AA	61	18(30)	0.995	0.543–1.823	0.986		
*TGFBR1*: rs10819638							
CT or TT	91	26(29)	1.000				
CC	54	18(33)	1.555	0.832–2.907	0.166		
*TGFBR1*: rs6478974							
TA or AA	76	19(25)	1.000				
TT	69	25(36)	1.245	0.679–2.285	0.479		
*TGFBR1*: rs10733710							
GA or AA	62	23(37)	1.000				
GG	83	21(25)	0.644	0.350–1.185	0.157		
*ACVR2A*: rs1424954							
GA or GG	102	33(32)	1.000				
AA	42	11(26)	0.757	0.376–1.523	0.436		
*SMAD1*: rs11939979							
CA or CC	56	17(30)	1.000				
AA	86	26(30)	0.989	0.526–1.860	0.974		
*SMAD3*: rs4776342							
AG or AA	102	32(31)	1.000				
GG	43	12(28)	0.928	0.475–1.811	0.826		
*SMAD3*: rs12102171							
CT or TT	79	23(29)	1.000				
CC	66	21(32)	1.303	0.711–2.386	0.392		
*SMAD3*: rs6494633							
CT	31	9(29)	1.000				
CC	114	35(31)	0.737	0.340–1.594	0.437		
*SMAD3*: rs11632964							
TC or TT	84	27(32)	1.000				
CC	61	17(28)	0.736	1.395–1.372	0.335		
*SMAD3*: rs750766							
AG or AA	89	26(29)	1.000				
GG	55	18(33)	1.218	0.661–2.243	0.527		
*SMAD3*: rs4776343							
AG	13	5(38)	1.000				
GG	132	39(30)	0.780	0.304–2.001	0.605		
*SMAD3*: rs11071938							
TC or TT	63	18(29)	1.000				
CC	82	26(32)	1.274	0.696–2.331	0.432		
*SMAD4*: rs948588							
GA or AA	14	6(43)	1.000				
GG	131	38(29)	0.725	0.300–1.753	0.476		
*SMAD4*: rs12456284							
AG or GG	84	25(30)	1.000				
AA	59	18(31)	0.884	0.473–1.651	0.699		
*SMAD4*: rs7244227							
AG or GG	99	31(13)	1.000				
AA	44	13(30)	0.821	0.424–1.587	0.821		
*SMAD4*: rs12455792							
CT or TT	98	33(34)	1.000				
CC	45	11(24)	0.596	0.298–1.192	0.143		
*SMAD4*: rs12958604							
AG or GG	112	34(30)	1.000				
AA	33	10(30)	0.897	0.438–1.837	0.767		
*SMAD4*: rs10502913							
AG or AA	79	24(30)	1.000				
GG	66	20(30)	0.893	0.487–1.638		0.714	
*SMAD6*: rs12906898							
AG or AA	34	10(29)	1.000				
GG	109	34(31)	1.226	0.592–2.539		0.583	
*SMAD6*: rs4776318							
CA or AA	52	15(29)	1.000				
CC	92	29(32)	1.161	0.609–2.216		0.650	
*SMAD7*: rs7227023							
GA	7	3(43)	1.000				
GG	138	41(30)	0.774	0.232–2.590		0.678	
*SMAD8*: rs7333607							
AG	25	8(32)	1.000				
AA	116	36(31)	1.043	0.480–2.265		0.916	
*SMAD8*: rs511674							
GA	28	8(29)	1.000				
AA	117	36(31)	1.282	0.592–2.777		0.529	

NOTE. Multivariate analyses in this table were adjusted for Stage, Histology, Age, and Smoking status. Similar results were obtained when multivariate analyses were adjusted for all the factors listed in Table1 (data not shown).

Abbreviations: HR, hazard ratio; CI, confidence interval.

## Discussion

In this study, we systematically evaluated associations between a comprehensive panel of genetic variants in TGF-β signaling pathway genes and BM risk. We found that SNPs in *SMAD6*: rs12913975 GG or *INHBC*: rs4760259 TT were associated with the incidence of BM. To the best of our knowledge, this is the first evidence showing this association in patients with lung cancer. With validation, this test could be used as a predictive biomarker to identify patients at high risk of developing brain metastasis during the first 24 months after diagnosis.

One of the polymorphisms we found to be associated with BM risk was in *SMAD6*, which encodes a protein that localizes to both nuclei and cytoplasm. Smad6 and Smad7 act as “inhibitory” Smads, inhibiting TGF-β family signaling [21]. Induction of Smad6 and Smad7 expression by bone morphogenic protein and TGF-β signaling represents an auto-inhibitory feedback mechanism in the TGF-β pathway [21]. *SMAD6* is expressed in most human tissues, including the lung, but its function in tumorigenesis is not yet established. A previous retrospective study showed that *SMAD7* overexpression is linked with a reduced incidence of bone metastases from melanoma and breast cancer [22]. The structural similarity between SMAD6 and SMAD7 proteins suggests that both proteins may be involved in metastasis via similar mechanisms. Variants in *SMAD6* have been linked with prognosis in ovarian cancer [23], breast cancer and pancreatic carcinoma; polymorphism in *SMAD6* have also been linked with survival in NSCLC [24]. Metastases, especially brain metastases, is an important factor associated with poor prognosis, and SNPs in *SMAD6* may contribute to metastases, include CNS metastases.

We also found *INHBC*: rs4760259 polymorphisms to be associated with BM risk. The *INHBC* gene is located on human chromosome 12, region q13.1, and encodes a protein named βC, belonging to the inhibin subgroup. Inhibin and activin proteins, along with various growth and differentiation factors, Muellerian inhibiting substance, and other proteins, belong to the TGF-β superfamily. Activins have many effects on mesoderm formation [25], cell proliferation and apoptosis [26], branching morphogenesis [27], inflammation [28] and reproduction [29]. One α-subunit and four β-subunit isoforms (βA, βB, βCand βE) have been found in mammals and humans [30]. The activin α, βA, and βB subunits and their homo−/heterodimers have been well characterized; activin A (βAβA), for example, is a pleiotropic protein that affects apoptosis, cell-cycle control, angiogenesis and immune suppression [31]. The precise role of the βC subunit, however, is unclear. Activin βC subunit mRNA has been detected in rat and mouse lung, epididymis, testis, uterus, spleen, posterior pituitary, and adrenal gland, and in human ovary, testis, placenta, and prostate [31]. The activin βC subunit or its dimers may oppose the action of activin A. In one in vitro study, the activin βC subunit had a pro-apoptotic effect in liver cell lines. Furthermore, the activin βC subunit was downregulated in prostate and liver tumor cell lines [32]. Transfection of the activin βC subunit into the PC3 prostate cancer cell line results in decreased activin A levels [33]. A recent study showed polymorphisms in *INHBC* is associated with ovarian cancer risk [34]. Another study showed it to be strongly associated with survival in NSCLC [24]. It can be seen that activin βC subunit is associate with tumorigenesis and progress, and metastases is a important step in tumor progression which strongly associated with poor prognosis, therefore we can believe SNPs in *INHBC* may contribut to BM.

A single SNP often provides a modest or undetectable effect whereas the amplified effects of combined SNPs in the same pathway may enhance predictive power. We analyzed the association with BM in patients with both two genotypes. A clear and significant trend was evident for higher risk with the combined subgroups. These results suggest a cumulative influence by multiple genetic variants within the TGF-β signaling pathway were able to further enhance predictive power.

However, neither of the SNPs we identified as being linked with BM is located in the coding region, which suggest that these SNPs may not affect *SMAD6* or *INHBC* function directly but rather may change levels of gene expression through being located in regulatory regions or through linkage to other SNPs that affect gene activity. Further in vitro and in vivo functional studies are needed to confirm the functional significance of the identified *SMAD6* and *INHBC* SNPs.

Moreover, we found that 48% of patients with both high risk alleles do not have brain metastasis (**Table 4**), and 1.7% (1/60) of patients with brain metastasis do not carry either of the two high risk genotypes. As the heterogeneity and genetic complexity of NSCLC, we speculate that a few other factors and SNPs in other signaling pathways that regulate cell proliferation and migration may be also associated with the risk of BM. Future research are needed to further enhance predictive power.

PCI is considered part of standard therapy for limited-stage SCLC, as up to 80% of these patients develop BM [35], [36]. Slotman et al. conducted a trial of 286 patients with extensive-stage SCLC who were randomized to receive either PCI or no PCI. Survival at 1 year improved from 13.3 to 27.1% [6]. At this time, nearly all patients with SCLC should be offered PCI to reduce the chance of BM and improve overall survival. Currently, the intensity of prophylactic therapy for acute lymphoblastic leukemia is adjusted to the risk of central nervous system (CNS) relapse [37]. Prior randomized, controlled trials [7], [8], [9] and several prospective studies evaluating PCI for NSCLC have been published. Studies have significantly show that PCI administered to patients improves intracranial disease control. However, none of these studies have ever shown a survival benefit with the application of PCI. The most recent RTOG trial (0214) evaluating the role of PCI in LA-NSCLC unfortunately closed early due to poor accrual. While there was a promising outcome in decreased intracranial metastases in the treatment group, this failed to result in a survival benefit [4]. Joseph A et al. analyzes it is unclear as to whether this is secondary to failure of identifying the cohort best suited for prevention, the inability of radiation to effect sufficient intracranial disease prevention because of a relatively radio-resistent tumor, or the need for more effective systemic therapies to control extracranial disease so that patients’ survival is long enough to see the benefit of PCI [1]. Because there is no predictive test to identify patients with high risk of brain metastatic, PCI has been given unselectively to all patients. PCI could negatively affect neurocognitive function and quality of life in those patients who do not need PCI [4]. If the findings from current study are validated in a study with adequate statistical power prospectively, we could use the SNPs identified in this study as a pretreatment test to select patients who would benefit from PCI, while avoiding PCI in patients who do not need it.

Our study had some limitations. The small number of patients raises the possibility that some of our findings were due to chance. Future studies are necessary to identity functional significance of the genetic variants we have identified, as well as to confirm or externally evaluate the associations in independent populations.

### Conclusions

We found that the GG genotype of *SMAD6*: rs12913975 and the TT genotype of *INHBC*: rs4760259 were associated with the incidence of BM in patients with NSCLC. These findings were confirmed in both Kaplan-Meier and multivariate Cox proportional hazard analyses, the latter adjusted for disease stage, tumor histology, age, and smoking status of the patient. These findings may be useful in future efforts to identify patients at high risk of brain metastasis.

## References

[1] Bovi JA, White J (2012) Radiation therapy in the prevention of brain metastases. Curr Oncol Rep 14: 55–62. 22134834 10.1007/s11912-011-0208-6

[2] Subramanian A, Harris A, Piggott K, Shieff C, Bradford R (2002) Metastasis to and from the central nervous system–the ‘relatively protected site’. Lancet Oncol 3: 498–507. 12147436 10.1016/s1470-2045(02)00819-7

[3] Nathoo N, Chahlavi A, Barnett GH, Toms SA (2005) Pathobiology of brain metastases. J Clin Pathol 58: 237–242. 15735152 10.1136/jcp.2003.013623 PMC1770599

[4] Gore EM, Bae K, Wong SJ, Sun A, Bonner JA, et al (2011) Phase III comparison of prophylactic cranial irradiation versus observation in patients with locally advanced non-small-cell lung cancer: primary analysis of radiation therapy oncology group study RTOG 0214. J Clin Oncol 29: 272–278. 21135270 10.1200/JCO.2010.29.1609 PMC3056462

[5] Gaspar LE, Scott C, Murray K, Curran W (2000) Validation of the RTOG recursive partitioning analysis (RPA) classification for brain metastases. Int J Radiat Oncol Biol Phys 47: 1001–1006. 10863071 10.1016/s0360-3016(00)00547-2

[6] Slotman B, Faivre-Finn C, Kramer G, Rankin E, Snee M, et al (2007) Prophylactic cranial irradiation in extensive small-cell lung cancer. N Engl J Med 357: 664–672. 17699816 10.1056/NEJMoa071780

[7] Umsawasdi T, Valdivieso M, Chen TT, Barkley HT Jr, Booser DJ, et al (1984) Role of elective brain irradiation during combined chemoradiotherapy for limited disease non-small cell lung cancer. J Neurooncol 2: 253–259. 6389779 10.1007/BF00253278

[8] Cox JD, Stanley K, Petrovich Z, Paig C, Yesner R (1981) Cranial irradiation in cancer of the lung of all cell types. JAMA 245: 469–472. 7452872

[9] Russell AH, Pajak TE, Selim HM, Paradelo JC, Murray K, et al (1991) Prophylactic cranial irradiation for lung cancer patients at high risk for development of cerebral metastasis: results of a prospective randomized trial conducted by the Radiation Therapy Oncology Group. Int J Radiat Oncol Biol Phys 21: 637–643. 1651304 10.1016/0360-3016(91)90681-s

[10] Cox JD, Scott CB, Byhardt RW, Emami B, Russell AH, et al (1999) Addition of chemotherapy to radiation therapy alters failure patterns by cell type within non-small cell carcinoma of lung (NSCCL): analysis of radiation therapy oncology group (RTOG) trials. Int J Radiat Oncol Biol Phys 43: 505–509. 10078629 10.1016/s0360-3016(98)00429-5

[11] Ceresoli GL, Reni M, Chiesa G, Carretta A, Schipani S, et al (2002) Brain metastases in locally advanced nonsmall cell lung carcinoma after multimodality treatment: risk factors analysis. Cancer 95: 605–612. 12209754 10.1002/cncr.10687

[12] Robnett TJ, Machtay M, Stevenson JP, Algazy KM, Hahn SM (2001) Factors affecting the risk of brain metastases after definitive chemoradiation for locally advanced non-small-cell lung carcinoma. J Clin Oncol 19: 1344–1349. 11230477 10.1200/JCO.2001.19.5.1344

[13] Grinberg-Rashi H, Ofek E, Perelman M, Skarda J, Yaron P, et al (2009) The expression of three genes in primary non-small cell lung cancer is associated with metastatic spread to the brain. Clin Cancer Res 15: 1755–1761. 19190132 10.1158/1078-0432.CCR-08-2124

[14] Massague J (2008) TGFbeta in Cancer. Cell 134: 215–230. 18662538 10.1016/j.cell.2008.07.001 PMC3512574

[15] Javelaud D, Alexaki VI, Mauviel A (2008) Transforming growth factor-beta in cutaneous melanoma. Pigment Cell Melanoma Res 21: 123–132. 18426405 10.1111/j.1755-148X.2008.00450.x

[16] Nguyen DX, Bos PD, Massague J (2009) Metastasis: from dissemination to organ-specific colonization. Nat Rev Cancer 9: 274–284. 19308067 10.1038/nrc2622

[17] Meulmeester E, Ten Dijke P (2011) The dynamic roles of TGF-beta in cancer. J Pathol 223: 205–218. 20957627 10.1002/path.2785

[18] Khuri FR, Kim ES, Lee JJ, Winn RJ, Benner SE, et al (2001) The impact of smoking status, disease stage, and index tumor site on second primary tumor incidence and tumor recurrence in the head and neck retinoid chemoprevention trial. Cancer Epidemiol Biomarkers Prev 10: 823–829. 11489748

[19] Yuan X, Liao Z, Liu Z, Wang LE, Tucker SL, et al (2009) Single nucleotide polymorphism at rs1982073:T869C of the TGFbeta 1 gene is associated with the risk of radiation pneumonitis in patients with non-small-cell lung cancer treated with definitive radiotherapy. J Clin Oncol 27: 3370–3378. 19380441 10.1200/JCO.2008.20.6763 PMC4385796

[20] Hildebrandt MA, Yang H, Hung MC, Izzo JG, Huang M, et al (2009) Genetic variations in the PI3K/PTEN/AKT/mTOR pathway are associated with clinical outcomes in esophageal cancer patients treated with chemoradiotherapy. J Clin Oncol 27: 857–871. 19164214 10.1200/JCO.2008.17.6297 PMC2738430

[21] Derynck R, Zhang YE (2003) Smad-dependent and Smad-independent pathways in TGF-beta family signalling. Nature 425: 577–584. 14534577 10.1038/nature02006

[22] Javelaud D, Alexaki VI, Dennler S, Mohammad KS, Guise TA, et al (2011) TGF-beta/SMAD/GLI2 signaling axis in cancer progression and metastasis. Cancer Res 71: 5606–5610. 21862631 10.1158/0008-5472.CAN-11-1194 PMC3165102

[23] Le Page C, Puiffe ML, Meunier L, Zietarska M, de Ladurantaye M, et al (2009) BMP-2 signaling in ovarian cancer and its association with poor prognosis. J Ovarian Res 2: 4. 19366455 10.1186/1757-2215-2-4 PMC2674440

[24] Lin M, Stewart DJ, Spitz MR, Hildebrandt MA, Lu C, et al (2011) Genetic variations in the transforming growth factor-beta pathway as predictors of survival in advanced non-small cell lung cancer. Carcinogenesis 32: 1050–1056. 21515830 10.1093/carcin/bgr067 PMC3128559

[25] McDowell N, Gurdon JB (1999) Activin as a morphogen in Xenopus mesoderm induction. Semin Cell Dev Biol 10: 311–317. 10441545 10.1006/scdb.1999.0307

[26] Hully JR, Chang L, Schwall RH, Widmer HR, Terrell TG, et al (1994) Induction of apoptosis in the murine liver with recombinant human activin A. Hepatology. 20: 854–862. 10.1002/hep.1840200413 7927226

[27] Ball EM, Risbridger GP (2001) Activins as regulators of branching morphogenesis. Dev Biol 238: 1–12. 11783989 10.1006/dbio.2001.0399

[28] Jones KL, de Kretser DM, Patella S, Phillips DJ (2004) Activin A and follistatin in systemic inflammation. Mol Cell Endocrinol 225: 119–125. 15451576 10.1016/j.mce.2004.07.010

[29] de Kretser DM, Hedger MP, Loveland KL, Phillips DJ (2002) Inhibins, activins and follistatin in reproduction. Hum Reprod Update 8: 529–541. 12498423 10.1093/humupd/8.6.529

[30] Xia Y, Schneyer AL (2009) The biology of activin: recent advances in structure, regulation and function. J Endocrinol 202: 1–12. 19273500 10.1677/JOE-08-0549 PMC2704481

[31] Butler CM, Gold EJ, Risbridger GP (2005) Should activin betaC be more than a fading snapshot in the activin/TGFbeta family album? Cytokine Growth Factor Rev 16: 377–385. 15925536 10.1016/j.cytogfr.2005.04.005

[32] Vejda S, Erlach N, Peter B, Drucker C, Rossmanith W, et al (2003) Expression of activins C and E induces apoptosis in human and rat hepatoma cells. Carcinogenesis 24: 1801–1809. 12949049 10.1093/carcin/bgg154

[33] Mellor SL, Ball EM, O’Connor AE, Ethier JF, Cranfield M, et al (2003) Activin betaC-subunit heterodimers provide a new mechanism of regulating activin levels in the prostate. Endocrinology 144: 4410–4419. 12960042 10.1210/en.2003-0225

[34] Yin J, Lu K, Lin J, Wu L, Hildebrandt MA, et al (2011) Genetic variants in TGF-beta pathway are associated with ovarian cancer risk. PLoS One 6: e25559. 21984931 10.1371/journal.pone.0025559 PMC3184159

[35] Auperin A, Arriagada R, Pignon JP, Le Pechoux C, Gregor A, et al (1999) Prophylactic cranial irradiation for patients with small-cell lung cancer in complete remission. Prophylactic Cranial Irradiation Overview Collaborative Group. N Engl J Med 341: 476–484. 10441603 10.1056/NEJM199908123410703

[36] Meert AP, Paesmans M, Berghmans T, Martin B, Mascaux C, et al (2001) Prophylactic cranial irradiation in small cell lung cancer: a systematic review of the literature with meta-analysis. BMC Cancer 1: 5. 11432756 10.1186/1471-2407-1-5 PMC34096

[37] Pui CH (2006) Central nervous system disease in acute lymphoblastic leukemia: prophylaxis and treatment. Hematology Am Soc Hematol Educ Program: 142–146. 10.1182/asheducation-2006.1.142 17124053

